# Apoptotic activity in Libyan breast cancer

**DOI:** 10.1186/1477-7819-10-102

**Published:** 2012-06-08

**Authors:** Jamela Boder, Fathi Abdalla, Mohamed Elfagieh, Abdelbaset Buhmeida, Yrjö Collan

**Affiliations:** 1 Department of Pathology, University of Turku, Henrikinkatu 10, Turku, 20014, Finland; 2 Department of Pathology, National Cancer Institute of Misurata, Misurata, Libya; 3 Department of Surgical Oncology, National Cancer Institute, Misurata, Libya; 4 Center of Excellence in Genomic Medicine Research, King Abdul-Aziz University, P.O. Box: 80216, Jeddah, 21589, Saudi Arabia

**Keywords:** AI, Standardized mitotic-apoptotic ratio, Libyan female BC, Survival, Clinicopathological features

## Abstract

**Background:**

We evaluated the relationship of the apoptotic activity index (AI) and the standardized mitotic-apoptotic ratio (SMI/AI) with clinicopathological features and prognosis in Libyan female breast cancer (BC) patients. We then compared our results with corresponding results in Finnish and Nigerian female BC patients.

**Methods:**

Histological samples of breast carcinoma from 130 patients were retrospectively studied: an estimation of the apoptotic activity per square millimeter (expressed as apoptotic activity index (AI)), and standardized mitotic-apoptotic ratio (SMI/AI) was made, and the results compared with the clinicopathological features and the patient’s survival.

**Results:**

There was a statistically significant correlation between the AI and most of the clinicopathological features; the strongest association was observed for clinical stage lymph node (LN) status (*P* = 0.005). There were also correlations between AI and histological grade (*P* = 0.035), large tumor size (*P* = 0.011) and the clinical stage (*P* = 0.009). There were, however, prominent AI differences between Libyan, Nigerian and Finnish populations. The mean values of AI and SMI/AI in Libyan BC patients were 12.8 apoptotic figures per square millimeter and 2.8, respectively. The Libyan AI is slightly higher than in Nigeria, but much higher than in Finland. The differences between countries are seen throughout the samples as well as being present in certain subgroups. The survival analysis indicated that short survival time was associated with high apoptotic indices values and so can identify aggressive tumors and provide significant prognostic support. The cutoff (4 and 18 apoptosis/mm^2^) of AI might be applied as a quantitative criterion for Libyan BC to separate the patients into good, moderate and bad prognosis groups.

**Conclusions:**

The results indicated that the differences in AI among the three countries may be due to the known variation in the distribution of genetic markers in these populations. Improvement in health care and introduction of screening programs, however, could be very helpful in the Libyan population.

## Background

Apoptosis or programmed cell death is the carefully coordinated collapse and death of the cell, associated with nuclear DNA fragmentation and protein degradation, usually followed by rapid engulfment of the remaining nuclear material by neighboring cells. It is an essential part of life for every multicellular organism. Apoptosis plays a major role throughout life, from embryonic development to senescence. Apoptosis is needed to destroy cells infected with viruses, cells with DNA damage, and cancer cells. Several oncogenes, such as Bcl-2 [1], c-myc [2] and p53 [3] are involved in the regulation of apoptosis. The role of apoptosis in oncogenesis is currently being studied intensively in breast cancer (BC). There are several methods to detect apoptosis.

Immunohistochemistry can detect death receptors and ligands, but also morphologic identification through microscopy is possible. Apoptotic count has predictive and prognostic roles in survival in BC in Caucasian women [4]. Apoptosis count has been shown to be a prognosticator in African BC [5]. Earlier studies have shown significant differences, at a population level, of proliferative activity and nuclear morphometric features among Libyan, Finnish and Nigerian BC [6,7].

This study is designed to evaluate how these observations are reflected in the pattern of the apoptotic activity in Libyan BC. In addition, an attempt will be made to estimate the prognostic role of apoptotic activity in helping to identify the high-risk group of BC patients.

## Methods

### Clinicopathological features

The study was performed on paraffin-embedded samples from Libyan female BC patients. All cases were diagnosed at the Department of Pathology, African Oncology Institute, Sabratha, Libya, and Tripoli Medical Center, Tripoli, Libya during the years 2000 to 2006. Patients were excluded from this study on the basis of the following exclusion criteria: histopathology was done elsewhere than in the mentioned study centers, patient history, medical files or specimens were not found, the follow-up was less than three months, and paraffin blocks were not available for recutting. After exclusion of patients’ samples, 130 patients remained in the study. A total of 115 patients were treated with modified radical mastectomy with axillary clearance. Fifteen patients were unfit for surgery due to distant metastases. Diagnostic biopsies were used for this study, a detailed history and clinicopathological features (age, menopausal status, tumor size, stage and grade, and lymph node status) were collected from patient files (Tables 1 and 2). The mean age at the time of diagnosis was 46.5 (SD ± 13.4) years; 4.6%, 33.8%, 49.2% and 12.3% of patients were at stages 1, 2, 3, and 4, respectively.

**Table 1:** Average estimates of apoptotic activity, and balance between proliferative and apoptotic activity in BCs, in different subgroups of 130 Libyan female BC patients

** Group**	**Number of patients**	**AI (SD)**	***P*value**	**SMI/AI (SD)**	***P*value**
Whole material	130	12.8 (9.6)		2.8 (2.0)	
Age groups					
<40	49	13.3 (9.5)		2.9 (2.2)	
40-49	32	9.9 (9.9)	0.12	2.4 (1.5)	0.38
≥50	49	14.3 (9.4)		3.0 (2.0)	
Menopausal status					
Premenopausal	80	12.07 (9.7)		2.76 (2.0)	0.54
(age <50 yrs)			0.26		
Postmenopausal	50	14.05 (9.5)		2.98 (2.0)	
(age ≥ 50 yrs)					
Tumor size					
<3	8	4.5 (5.7)	0.011	2.8 (1.9)	0.75
≥3	122	13.4 ( 9.6)	3.2	(2.2)	
Lymph node (LN) status					
LN-	27	8.3 (7.0)	0.005	2.5 (1.3)	0.73
LN+	103	14.0 (9.9)		2.9 (2.1)	
Clinical stage					
Stage1	6	5.0 (5.1)	3.0 (2.3)		
Stage 2	44	10.2 (6.7)	0.009	2.4 (1.0)	0.30
Stage 3	64	14.5 (10.5)		2.9 (1.9)	
Stage 4	16	16.4 (11.4)		3.4 (0.8)	
Histological grade					
1	10	5.9 (5.7)	0.035	2.0 (1.1)	0.43
2	70	12.7 (7.4)		3.0 (2.1)	
3	50	14.5 (9.0)		2.8 (1.8)	
Histological type					
Invasive ductal	95	13.3 (10.6)	0.61	2.9 (2.0)	0.61
Invasive lobular	13	10.8 (5.8)		3.2 (2.5)	
Other carcinomas	22	12.0 (6.7)		2.8 (2.0)	

*AI* apoptotic activity index, *BC* breast cancer, *SMI/AI* standardized mitotic-apoptotic ratio, *SD* standard deviation.

**Table 2:** Mean estimates of apoptotic activity in different TNM and clinical stages of 130 Libyan female BC patients, (*T= diameter of tumor, **N = lymph node status, ***M= distant metastasis; present or absent)

** Group**	**Number of patients**	**AI (SD)**	***P*value**	**SMI/AI (SD)**	***P*value**
*T Stage			0.007		0.06
T1	6	4.0 (5.5)		3.4 (2.6)	
T2	45	10.4 (6.8)		2.5 (1.2)	
T3	44	15.5 (10.7)		2.8 (2.0)	
T4	35	14.1 (10.6)		3.3 (2.6)	
**N Stage			0.007		0.02
N0	27	8.3 (7.0)		2.5 (1.3)	
N1	69	12.3 (8.2)		3.0 (2.0)	
N2	32	17.0 (12.2)		2.7 (2.2)	
N3	2	26.0 (4.6)		2.4 (1.4)	
***M Stage			0.02		0.013
M0	114	12.3 (9.3)		2.7 (1.6)	
M1	16	16.4 (11.4)		4.0 (3.4)	
Clinical stage					
Stage 1	6	5.0 (5.1)		3.0 (2.3)	
Stage 2	44	10.2 (6.7)	0.009	2.4 (1.0)	0.30
Stage 3	64	14.5 (10.5)		2.9 (1.9)	
Stage 4	16	16.4 (11.4)		3.4 (0.8)	

The significance of the correlation of stage features and proliferative features are shown as *P* values evaluated with ANOVA and *t* test. *T stage, diameter of the primary neoplasm; **N stage, lymph node status: extent of lymph node involvement; ***M stage, distant metastasis: present/absent. AI, apoptotic activity index; BC, breast cancer; SMI/AI, standardized mitotic-apoptotic ratio; SD, standard deviation.

The permission for tumor sample collection has been obtained from the Libyan National Authority for Medical Affairs. This study is a part of BC studies, permission for which has been obtained from the local ethics committee of National Cancer Institute in Misurata, Libya.

### Follow-up and treatment

The follow-up data were collected from patient files. Follow-up time ranged from four to seventy-eight months, Average follow-up was 32.9 months. Some patients were lost from follow-up. BC was recorded as the underlying cause of death for 34 patients. Three cases died of causes unrelated to BC and were not included in the survival analysis. No autopsies were performed. The survival period was defined as the time from diagnosis either to the time of death or to the date on which the patient was known to be alive.

### Histological methods

The tumor diameter was measured after surgical removal in three dimensions, then biopsy specimens were fixed in buffered formalin (pH 7.3), and embedded in paraffin. Sections of 5 μm thickness were stained with hematoxylin and eosin stain. The histological typing in our study was based on the International Histological Classification of Tumours 1981, and grading of tumors was done according the modified Bloom Richardson histopathological grading system. There were ninety-five invasive ductal carcinomas (73.1%), thirteen invasive lobular carcinomas (10%), seven mixed ductal and lobular carcinomas (5.4%), six medullary carcinomas (4.6%), three papillary carcinomas (2.3%), five mucinous carcinomas (3.8%), and one metaplastic carcinoma (0.8%).

In identifying the apoptotic bodies, we applied criteria described by van de Schepop *et al*. [8]. Apoptotic cells were characterized by shrunken acidophilic bodies with fragmented nuclei and condensed nuclear chromatin. The apoptotic cells were separated from their neighbors and lacked associated inflammatory reaction. Counting was carried out in the most cellular region at tumor periphery, avoiding areas of necrosis, inflammation, *in situ* carcinoma, and calcification. A five-week training program on apoptotic counting was based on a set of ten Libyan female BC samples. During that period, counts were repeated on ten separate occasions, two to four days apart. We used an Olympus laboratory microscope (x40, objective lens magnification, numerical aperture 0.75, field diameter, 490 μm; Olympus Corp, Tokyo, Japan). The number of apoptotic bodies in 10 consecutive fields from the most cellular area of the sample was the apoptotic activity (AA). The volume fraction-corrected AI or AI gives the apoptotic count as the number of apoptotic bodies by the area of the neoplastic tissue in the microscopic fields. This is the number of apoptosis in 10 consecutive fields corrected for the volume fraction and field size. In this method, the area fraction (as an estimate of volume fraction) of neoplastic tissue in the microscopic field is evaluated simultaneously with the apoptotic count [9,10]$AI=k\;(\frac{\sum AA}{\sum Vv})$, where k = 100\πr², r is the radius of the microscopic field, AA is the number of apoptotic bodies in the studied field and Vv is the volume fraction (estimated by the area fraction, as a percentage) of malignant epithelium in the studied field. The ratio of SMI and AI (SMI/AI) was also calculated to evaluate the balance between cell proliferation and cell death.

### Statistical analysis

The variables of the material were grouped into logical classes and descriptive statistics calculated for the continuous variables using SPSS 15.0 for Windows. For survival analysis, Kaplan-Meier curves were plotted, and differences between the curves analyzed using the logrank test. The AI thresholds were the cutoff points showing curve separation with the highest statistical significance. Univariate analysis was performed to evaluate the distribution of AI, and standardized mitotic-apoptotic ratio (SMI/AI) within clinicopathological factors. *P* values below 0.05 were regarded as significant. Student *t* tests and ANOVA were also used to test differences between the groups.

## Results

### Correlation of AI and SMI/AI with the clinicopathological features

**Figure 1:**
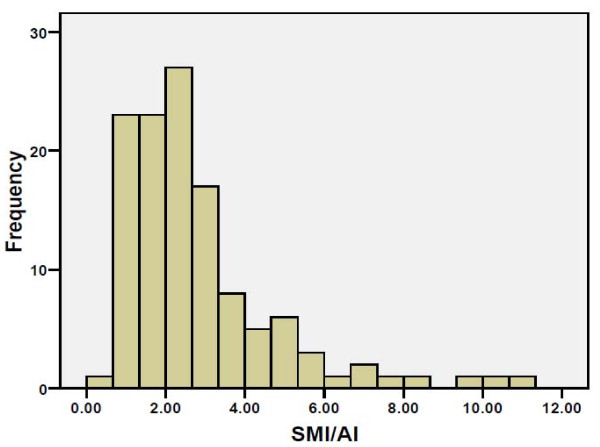
Distribution of SMI/AI values in 130 Libyan female BCs.

**Figure 2:**
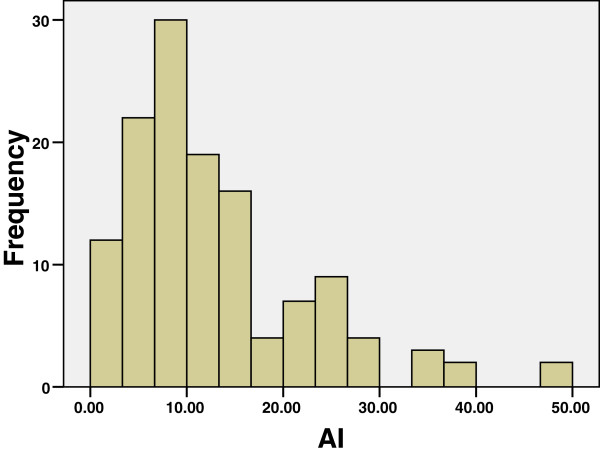
Distributions of AI in 130 cases of Libyan BC.

**Figure 3:**
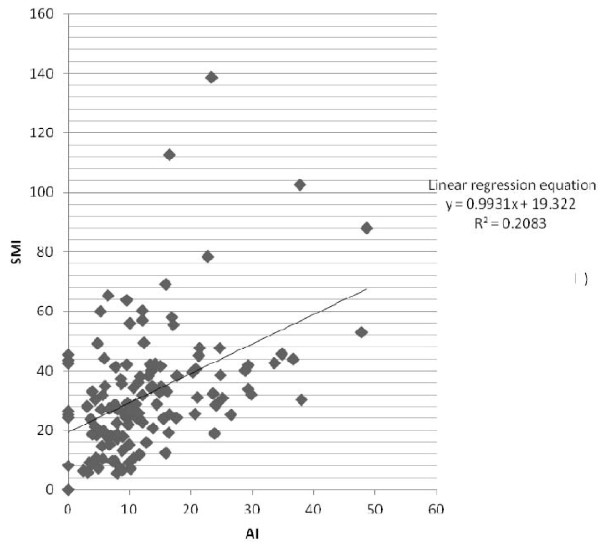
**Correlation between SMI and AI in 130 Libyan female BCs.** Clearly, there is a correlation (*P* <0.001), but the correlation coefficient is low (r = 0.193).

The clinical characteristics of the Libyan female BC patients (n = 130) and average estimate of AI and SMI/AI ratio are described in Tables 1 and 2. The distributions of the values are shown in Figures 1 and 2. The relationship of SMI and AI is shown in Figure 3. Clearly, AI is much smaller than SMI. The regression line shows there is a positive relationship between SMI and AI with r = 0.446, *P* < 0.001 The AI in the whole material, and in different subgroups defined by the menopausal status, lymph node status, tumor size, histological grade, histological type, clinical stage, are shown in Table 2, and TNM stage in Table 3. The highest significance of correlation is shown between clinical lymph node status and the AI (*P* = 0.005). Higher values also seen in large tumors, and those of higher histological grade and advanced clinical stage with *P* values 0.011, 0.035 and 0.009, respectively. The difference in the AI between invasive ductal carcinoma and lobular carcinoma was statistically insignificant (*P* = 0.35) (Table 3). However, SMI/AI does not show a significant relationship with histopathological features.

**Table 3:** Average estimates of apoptotic activity among 130 Libyan female breast cancer patients

** Group**	**Number**	**AI (SD) Nigeria**	***P*value**	**Number**	**AI (SD) Finland**	***P*value**	**Number**	**AI (SD) Libya**	***P*value**
Whole material	300	9.6 (14.8)		285	5.2 (6.1)		130	12.8 (9.6)	
Menopausal status									
Premenopausal	223	10.1 (16.3)	0.333	93	5.6 (6.4)	0.44	80	12.07 (9.7)	0.26
Postmenopausal	77	8.2 (9.3)		192	5.0 (6.0)		50	14.05 (9.5)	
Lymph node status									
LN-	65	7.6 (10.6)	0.185	188	5.3 (6.8)	0.514	27	8.3(7.0)	0.005
LN+	235	10.2 (15.7)		97	4.8 (4.8)		103	14.0 (9.9)	
Histological grade									
1	44	4.0 (7.1)		67	2.9 (4.8)		10	5.9 (5.7)	
2	119	8.6 (11.5)	0.012	173	4.5 (4.9)	<0.001	70	12.7 (7.4)	0.035
3	137	12.4 (19.8)		45	11.1 (8.4)		50	14.5 (9.0)	
Invasive ductal	242	10.3 (16.7)	0.070	231	5.9 (6.5)	0.017	95	13.3 (10.6)	0.35
Other carcinoma	58	5.3 (8.4)		54	2.1 (2.3)		35	11.5 (6.3)	
Stage									
1	65	7.6 (10.6)		95	4.5 (5.1)		6	5.0 (5.1)	
2	75	10.1 (11.7)	0.185	171	5.7 (6.9)	0.139	44	10.2 (6.7)	0.009
3	98	10.2 (20.3)		19	4.1 (2.7)		64	14.5 (10.5)	
4	62	10.2 (11.4)		*NA	NA		16	16.4 (11.4)	

Results are compared with Finnish and Nigerian patients in different subgroups. *P* value gives the statistical significance of the relationship between apoptotic counts (AI) and characteristics of the patient groups (*t* test, X² test, ANOVA). *NA not available. *AI* apoptotic activity index: *SD* standard deviation.

### Quantitative relationship between SMI and AI

Surprisingly, the ratio of SMI/AI in Libyan material is less than previous work on Nigerian material and close to the results on Finnish material.

### Correlation of AI with the survival outcome

**Figure 4:**
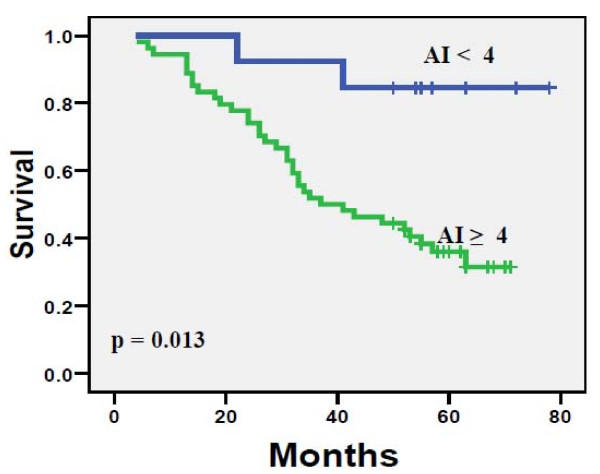
**Disease-specific survival (DSS) for 130 Libyan patients with BC divided according to AI cut point of four.** The difference between the curves is highly significant (logrank *P* = 0.013).

**Figure 5:**
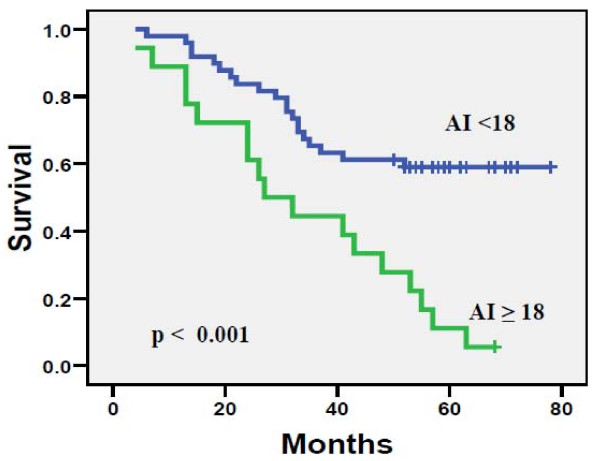
**Disease-specific survival (DSS) for 130 Libyan patients with BC divided according to AI cut point of 18.** The difference between the curves is highly significant (logrank *P* = 0.001).

AI was a significant predictor of disease-specific survival (DSS) in the overall material and was negatively correlated with survival time. The analysis for determination of decision cut points in the Libyan material resulted in an obvious two cutoff points (at four, and eighteen) surrounded by less significant cut points. The analysis using Kaplan-Meier curves of AI indicated that short survival time was correlated with high apoptotic activity tumors (Figures 4 and 5).

## Discussion

This study is a continuation of our efforts to further elucidate the biology of Libyan BC and to compare our results with the corresponding results of Finnish, and Nigerian female BC patients and also to identify more effective prognostic factors than the traditional staging system to aid therapeutic decision making. The aim of the present study was to cast further light on the issues related to prognostication of BC, while assessing the value of apoptotic activity as an independent prognostic factor. In this study, we focused on stage I to IV disease, where molecular and other markers may help pinpointing a subgroup of patients who would eventually benefit from the use of adjuvant therapy for their disease. This important decision involves a careful weighing of the risks of toxicity and complications against the potential curability of the disease [11]. It is well established that early BC can be cured with radical surgical resection [12].

Unfortunately, however, 20% to 30% of the patients with lymph node-negative (LN-) BC die of recurrent disease. Prediction of disease outcome in individual patients after curative resection is still far from reliable [13]. However, there is some hope and our results already suggest that counting of apoptotic body could be used to help in this decision. Apoptosis has been recognized as one of the features of BC cells that distinguish them from normal cells, because it plays an important part in tumorigenesis and today many researches consider that induction of apoptosis can be useful as a potential therapeutic target [14,15]. In addition, more rational decisions can be made as soon as we learn more of markers and diagnostic tools in accurate prediction of the disease outcome in individual patients [16,17].

On the basis of the present results, we believe that this detection can be improved using the assessment of apoptotic count in the primary tumors, and in this assessment, quantifying both the AA and AI seem to be very important. In this study, we compared the two methods for counting apoptosis, and found both of them significantly provide the most consistent and meaningful correlations to the clinicopathological variables. In the present cohort, several interesting and important observations were made, all implicating that the quantitatively measurable apoptotic bodies of tumor cells could provide significant prognostic information in BC.

First, a significant correlation was shown between apoptotic count and tumor grade. Several studies concluded that apoptosis is strongly correlated to the histological grade of ductal carcinoma, in that poorly differentiated tumors show higher AI than well differentiated tumors [1,18,19]. Our results were consistent with these findings.

With regard to LN involvement, a trend with high significance was observed between AI and LN involvement; 79% of the tumors with LN involvement showed high AI. The preferential high AI in LN metastases is consistent with the view of clonal selection of tumor cells with apoptotic activity, conferring them a growth advantage with a higher potential of metastasis. This suggests that tumors with higher AI might be more aggressive and more likely to be associated with LN involvement at diagnosis, and was associated with shorter disease-specific survival. Many studies [19,20] also reported a similar significant correlation between high AI and axillary lymph node involvement and correlated them with the number of involved nodes.

Therefore, AI seems to be an indicator of poor prognosis in BC as well and was shown to be correlated with tumor stage. Our present observations are fully consistent with a previous report by Zhang *et al.*[19], confirming that 62% of the patients with advanced disease (stages III and IV) have higher AI tumors. Furthermore, AI in stages III and IV tumors was significantly higher than in stages I and II disease (*P* = 0.009), implicating a direct relationship between AI and the stage of the disease; higher AI is associated with more advanced stage and propensity to develop metastatic disease.

However, Lipponen *et al*. [9] did not show same trend. Part of these variations and discrepant observations might be explained by the inconsistencies in the methodological technical aspects of the measurement of the AI, which may refer to the number of apoptotic cells per square millimeter of neoplastic tissue in the section [5,10] or the percentage of cells in the section that are apoptotic [1,21]. In addition, it is well known that aggressive tumors consist of large areas of necrosis that may affect the identification of apoptotic cells in conventional sections, especially when the tumor is also heavily infiltrated by inflammatory cells. Special staining techniques may identify cells that contain fragmented DNA as a result of causes not related to apoptotic cell death. Moreover, introducing of some other quantitative tools, such as immunohistochemical staining and RT-PCR, could help in overcoming this problem, by finding biological markers that are more reliable to detect the apoptosis on a molecular basis.

Interestingly, 16% of our patients had metastasis at diagnosis, strengthening the hypothesis that BC is a systemic disease from presentation [22]. Most of these patients showed higher AI than others with local disease. This is in support of the hypothesis of Mommers *et al.*[23] who suggested that proliferation is more important than apoptosis in the transformation from preinvasive carcinoma to invasive type. On the other hand, apoptosis may play a major role in the advanced stages of BC progression.

Obviously, one of the most important observations of the present study is the one linking AI with the disease outcome, that is, the appearance of recurrence and long-term disease-specific survival (DSS). This is clinically relevant because a number of BC patients of different stages are at high risk of recurrence, it would be of paramount importance to develop reliable markers that would accurately predict those patients to be considered for adjuvant therapy. In univariate (Kaplan-Meier) survival analysis, AI was also a significant predictor of DSS, and not unexpectedly, AI was more often higher in patients who eventually died of their disease as compared with those who were alive at the completion of the follow-up, and this difference was significant (*P* = 0.001, Figure 5). These data clearly implicate that BC with high AI is at high risk for local or distant recurrence and, because of the high adverse prognostic impact of disease recurrence; these patients are also more likely to die of their disease [20]. To avoid this, these patients should be appropriate candidates for intensive follow-up and targeted therapeutic strategy.

Interestingly, although our study used similar methodology of the earlier study of Ikpatt *et al.*[24], it showed significance differences between BC in Libya compared with the Nigeria and Finland population. There was a significant difference in the whole material. The apoptotic and proliferative differences between Central African, North African and European patients may partly be related to biological differences and variation in genetic marker distribution between Central and North African, and European populations [25]. Our study has also shown difference between Libyan and Nigerian subgroups, further strengthening our suggestion of biological differences between North and sub-Saharan African populations. Studies of ethnic differences in BC pathological features are limited, except for those between African American women, and Caucasian American women. African American women are known to have aggressive BC compared with Caucasian American women [26,27].

Interestingly, our quantitative analysis of both SMI and AI values demonstrates strong correlation (Figure 3). This quantitative relationship between SMI and AI has also been described by several authors [1,9,10,24]. The regression analysis has been suggested that SMI is the most important determinant of AI [9]. Other markers for proliferation such as Ki-67 [1,21], also have been found to be highly significantly related to AI. Our finding shows that standard mitotic-apoptotic ratio is low, which is near to the results in Finnish patients, and that is because the mitotic and apoptotic activities are almost equal in BC, and this is in consistent with other findings [24,28].

## Conclusions

The results indicate that the differences in AI among the three countries studied may be due to the known variation in the distribution of genetic markers in these populations. Improvement in health care and introduction of screening programs, however, could be very helpful in the Libyan population and specific cutoff points (of 4 and 18 apoptosis/mm^2^) of AI might be used as a quantitative criterion for Libyan BC to separate the patients into good, moderate and bad prognosis groups.

## Competing interests

The authors declare that they have no competing interests.

## Authors’ contributions

JB performed research, counted the apoptotic figures and contributed to the design and preparation of the manuscript. FA statistically analyzed the data. ME provided the samples and clinical data. AB and YC designed and coordinated the research and drafted the manuscript. All authors read and approved the final manuscript.
